# Anti-NMDA Receptor Autoantibody Is an Independent Predictor of Hospital Mortality but Not Brain Dysfunction in Septic Patients

**DOI:** 10.3389/fneur.2019.00221

**Published:** 2019-03-15

**Authors:** Hamilton Malfussi, Iara Vidigal Santana, Juciano Gasparotto, Cassia Righy, Cristiane Damiani Tomasi, Daniel Pens Gelain, Fernando A. Bozza, Roger Walz, Felipe Dal-Pizzol, Cristiane Ritter

**Affiliations:** ^1^Programa de Pós-Graduação em Ciências Médicas, Universidade Federal de Santa Catarina, Florianópolis, Brazil; ^2^Laboratório de Fisiopatologia Experimental, Universidade do Extremo Sul Catarinense, Criciúma, Brazil; ^3^Departamento de Bioquímica, Centro de Estudos em Estresse Oxidativo, Instituto de Ciências Básicas da Saúde, Universidade Federal do Rio Grande do Sul, Porto Alegre, Brazil; ^4^Instituto Estadual do Cérebro Paulo Niemeyer, Rio de Janeiro, Brazil; ^5^Instituto Nacional de Infectologia Evandro Chagas, Fundação Oswaldo Cruz, Rio de Janeiro, Brazil; ^6^Grupo de Pesquisa em Gestão do Cuidado, Integralidade e Educação na Saúde (GECIES) – Programa de Pós-Graduação em Saúde Coletiva, Universidade do Extremo Sul Catarinense, Criciúma, Brazil; ^7^Instituto D'Or de Pesquisa e Ensino (IDOR), Rua Diniz Cordeiro, Rio de Janeiro, Brazil; ^8^Serviço de Neurologia, Departamento de Clínica Médica, Centro de Cirurgia de Epilepsia de Santa Catarina (CEPESC), Centro de Neurociências Aplicadas (CeNAp), Hospital Universitário (HU), Universidade Federal de Santa Catarina (UFSC), Florianópolis, Brazil; ^9^Hospital São José, Criciúma, Brazil

**Keywords:** delirium, brain dysfunction, sepsis, neuronal autoantibodies, ICU

## Abstract

The presence of autoantibodies against neuronal cell surface or synaptic proteins and their relationship to autoimmune encephalitis have recently been characterized. These autoantibodies have been also reported in other pathologic conditions; however, their role during sepsis is not known. This study detected the presence of autoantibodies against neuronal cell surface or synaptic proteins in the serum of septic patients and determined their relationship to the occurrence of brain dysfunction and mortality. This prospective, observational cohort study was performed in four Brazilian intensive care units (ICUs). Sixty patients with community-acquired severe sepsis or septic shock admitted to the ICU were included. Blood samples were collected from patients within 24 h of ICU admission. Antibodies to six neuronal proteins were assessed, including glutamate receptors (types NMDA, AMPA1, and AMPA2); voltage-gated potassium channel complex (VGKC) proteins, leucine-rich glioma-inactivated protein 1 (LGI1), and contactin-associated protein-2 (Caspr2), as well as the GABAB1 receptor. There was no independent association between any of the measured autoantibodies and the occurrence of brain dysfunction (delirium or coma). However, there was an independent and significant relationship between anti-NMDAR fluorescence intensity and hospital mortality. In conclusion, anti-NMDAR was independently associated with hospital mortality but none of the measured antibodies were associated with brain dysfunction in septic patients.

## Introduction

The presence of autoantibodies against neuronal cell surface or synaptic proteins and their relationship with autoimmune encephalitis have recently been characterized (1). Anti-N-methyl-D-aspartate (NMDA) receptor (NMDAR) was the first to be reported; since then, different types of autoantibodies against neuronal cell surface or synaptic proteins have been described (2). There are currently 16 known disorders with autoantibodies against cell surface or synaptic proteins (2). These autoantibodies were originally described in association with different tumors, mainly in young women (3–5).

These autoantibodies have been also reported in other pathologic conditions such as systemic lupus erythematous, epilepsy, stroke, mania, and schizophrenia in up to 30% of patients (6–12). Additionally, the presence of anti-NMDAR antibodies had been associated with worse neurologic outcomes after cardiac surgery (13). The functional consequences of anti-NMDAR have been characterized (14–17). They include the internalization of NMDARs in both excitatory and inhibitory hippocampal neurons and the induction of reduced NMDAR-mediated synaptic currents (14–17). Mice infused with cerebrospinal fluid (CSF) from auto-immune encephalitis patients showed memory deficits and depressive behaviors (18), as well as alterations in long-term potentiation (19), which are compatible with the proposed modifications on NMDA-mediated synaptic currents. Additionally, these autoantibodies also activate microglial cells (20) and their effects upon brain function appear to be related to alterations in the permeability of the blood-brain barrier (BBB) (21).

The induction of autoantibodies occurs rapidly in sepsis and in some cases remained elevated for several weeks (22). Antibodies have been identified against a spectrum of autoantigens including potassium channel regulator, gastric ATPase, glutamic decarboxylase-65, and several cytokines (22). In this context, therefore, autoantibodies against neuronal cell surface or synaptic proteins could be prevalent in septic patients, contributing to sepsis-associated brain dysfunction. Thus, the aim of the present study was to detect the presence of different autoantibodies against neuronal cell surface or synaptic proteins in serum of septic patients and to determine their relationship with the occurrence of brain dysfunction and mortality.

## Methods

This study was conducted according to the principles of the Declaration of Helsinki; in addition, the Ethics Committee of D'Or Institute, Rio de Janeiro and São José Hospital, Criciúma approved the study and all patients or their proxies provided written informed consent.

From August 2015 to May 2016, all consecutive adult (between 18 and 85 years of age) patients admitted to four different intensive care units (ICUs) (three in Rio de Janeiro and one in Criciúma, Brazil) with a diagnosis of community-acquired severe sepsis or septic shock were prospectively followed. Severe sepsis and septic shock were defined according to the 2001 SCCM/ESICM/ACCP/ATS/SIS International Sepsis Definitions Conference (23). Moribund patients (expected to die within 48 h) or those admitted to the ICU for palliative care, patients expected to stay in the ICU for <48 h, deafness, and an inability to speak Portuguese, as well as patients with a history of neurologic disorders, were excluded. No patient had a clinical suspicion or laboratory abnormalities associated with autoimmune encephalitis.

A random sample of the original cohort (40 patients from Rio de Janeiro and 20 patients from Criciúma) was analyzed. Blood was collected from these patients within 24 h of ICU admission to measure the levels of serum autoantibodies. Autoantibody levels were also measured in four young, healthy individuals.

## Definitions, Participant Selection, and Data Collection

Demographic, clinical, and laboratory data were collected using standardized case report forms that included the main diagnosis for ICU admission, comorbidities, Simplified Acute Physiology Score (SAPS) II (24), and Sequential Organ Failure Assessment (SOFA) score (25). The duration of mechanical ventilation (MV) and the use of sedatives were also obtained. Patients were assessed for delirium twice daily by a trained investigator during the first 14 days of ICU stay. The level of arousal was measured using the Richmond Agitation Sedation Scale (RASS) (26). Coma was defined as RASS scores of−4 (responsive only to physical stimulus) or−5 (unresponsive to physical stimulus) (27). Delirium was diagnosed using the Confusion Assessment Method (CAM)-ICU (28). Brain dysfunction was defined as the presence of coma and/or delirium. All ICUs had a protocol for the daily suspension of sedation in order to perform RASS and CAM-ICU assessments.

## Autoantibody Detection

Serum levels of antibodies to the following six neuronal proteins were assessed using the Autoimmune Encephalitis Mosaic 1 kit (EUROIMMUN, Luebeck, Germany): glutamate receptors (type NMDA, α-amino-3-hydroxy-5-methyl-4-isoxazolepropionic acid - AMPA1, and AMPA2); voltage-gated potassium channel complex proteins -leucine-rich glioma-inactivated protein 1 (LGI1), and contactin-associated protein-2 (Caspr2); as well as gamma-aminobutyric acid (GABA) B1 receptor. Briefly, the diluted serum samples were applied to the reaction fields and the reaction started by fitting the BIOCHIP slides into the reagent tray. The slides were then incubated with labeled antibody, drops of embedding medium were added to the BIOCHIP slides, and a cover glass was fitted. Images were acquired using a Microscopy EVOS® FL Auto Imaging System (AMAFD1000—Thermo Fisher Scientific; MA, USA) at 20x magnification. Fluorescence quantification was performed using ImageJ software (ImageJ v1.49, National Institutes of Health, USA). The fluorescence intensity was measured and expressed in arbitrary units.

## Data Processing and Statistical analysis

Standard descriptive statistics were produced and continuous variables were reported as means and standard deviation (SD). Comparisons between groups were performed using two-tailed Student's t- or Mann-Whitney U tests for continuous variables according to the data distributions. Fisher's exact and chi-square tests were used for comparisons between categorical variables, as appropriate.

Due to the small number of events, the independent association between autoantibodies and brain dysfunction was analyzed in a regression model that included only variables with *p* < 0.05 in univariate analysis. Since age is incorporated in the SAPS II score, models including SAPS II did not also include patient age. This was also true of the SOFA score since several aspects of the score are incorporated in the SAPS II; thus, when SAPS II was included in the regression, the SOFA was not. Since there is a co-linearity between sedation use and mechanical ventilation, only sedation use was incorporated in the model.

Regression models were also constructed to evaluate the association between autoantibodies and hospital mortality. The same exclusions cited to the brain dysfunction model also applied in the mortality model. However, the mortality models also considered the co-linearity between sedation, mechanical ventilation, and brain dysfunction, and included only brain dysfunction in the model.

Statistical significance was set at *p* < 0.05. All analyses were performed using IBM SPSS Statistics for Windows, version 23.0 (IBM Corp., Armonk, NY, USA).

## Results

The majority of patients included in the current study presented septic shock and predominant infection site was the lung. As expected the prevalence of brain dysfunction was high in this population. Sixty-eight percent of the population presented brain dysfunction (coma and/or delirium) during their ICU stay. Half of the included patients experienced coma and 52% experienced delirium. No healthy volunteer (n = 4) was positive for any of the measured autoantibodies. Supplementary Figure 1 shows representative images of the fluorescence of the autoantibodies.

The association between brain dysfunction and autoantibodies is shown in Table 1. There was no consistent correlation between the fluorescence intensity of the of autoantibodies and brain dysfunction in septic patients, except for a lower intensity of anti-NMDAR in comatose patients and a lower intensity of anti-GABAR in delirium patients. However, the regression analysis revealed no independent association between the intensity of autoantibodies fluorescence and brain dysfunction.

**Table 1:** Predictors of any brain dysfunction, delirium, or coma.

**Variable**	**Brain dysfunction**		**Delirium**		**Coma**	
	**Yes (*n* = 41)**	**No (*n* = 19)**	**Yes (*n* = 31)**	**No (*n*=29)**	**Yes (*n* = 30)**	**No (*n* = 30)**
Age, years, mean (SD)	71 (17)	64 (17)	74 (15)	63(18)^*^	66(17)	70 (18)
SAPS II, mean (SD)	67 (9)	55 (15)^*^	65 (14)	62 (19)	54 (14)	73 (15)^*^
SOFA, mean (SD)	7.1 (4.5)	3.6 (4)^*^	6.7 (4.2)	5.2 (5)	7.7 (4.4)	4.2 (4.2)^*^
Gender, male, number (%)	28 (68)	6 (32) ^*^	24 (70)	10 (34)^*^	20 (66)	14 (47)
Sedation, yes, number (%)	35 (85)	3 (16)^*^	25 (81)	13 (45)^*^	30 (100)	8 (27)^*^
Mechanical ventilation, yes, number (%)	34 (83)	2 (11)^*^	24 (77)	12 (41)^*^	30 (100)	6 (20)^*^
Septic shock, yes, number (%)	30 (73)	15 (79)	20 (64)	25 (86)	27 (90)	18 (60)^*^
Anti-NDMAR, mean (SD)	63 (17)	62 (20)	66 (16)	59 (19)	58 (15)	67 (19) ^*^
Anti-LGI1, mean (SD)	32 (26)	26 (13)	26 (14)	30 (21)	24 (11)	32 (22)
Anti-CASPR2, mean (SD)	67 (23)	58 (16)	58 (17)	64 (20)	57 (15)	66 (21)
Anti-GABABR, mean (SD)	16 (25)	9 (17)	7 (17)	16 (22)^*^	7 (10)	16 (25)
Anti-AMPAR1, mean (SD)	68 (20)	65 (18)	64 (19)	68 (18)	67 (19)	65 (18)
Anti-AMPAR2, mean (SD)	10 (22)	3 (6)	2 (3)	9 (19)	3 (6)	7 (18)

* *p < 0.05*.

The association between autoantibodies and ICU and hospital mortality is shown in Table 2. There was no significant association between autoantibodies and ICU mortality. However, there was a significant association between the fluorescence of NMDAR, LGI1, CASPR2, and AMPAR1 and hospital mortality. From all these antibodies, only anti-NMDAR was independently associated with hospital mortality in the regression analysis. A lower fluorescence intensity was associated with hospital mortality.

**Table 2:** Prediction of ICU and hospital mortality.

**Variables**		**ICU mortality**		**Hospital mortality**	
		**Yes (*n* = 17)**	**No (*n* = 43)**	**Yes (*n* = 27)**	**No (*n* = 33)**
Age, years, mean (SD)		68 (21)	69 (16)	73 (19)	64 (15)^*^
SAPS II, mean (SD)		62 (17)	68 (15)	61 (16)	66 (17)
SOFA, mean (SD)		6 (4.8)	5.7 (4.1)	5.6 (5.1)	6.4 (3.9)
Gender, male, number (%)		8 (47)	26 (60)	13 (48)	21 (64)
Sedation, yes, number (%)		15 (88)	23 (53)^*^	21 (78)	17 (52)^*^
Mechanical ventilation, yes, number (%)		15 (88)	21 (49)^*^	21 (78)	15 (45)
Septic shock, yes, number (%)		14 (82)	31 (72)	22 (81)	23 (70)
Brain dysfunction, yes, number (%)		16 (94)	25 (58)^*^	24 (89)	17 (52)^*^
Anti-NMDAR, mean (SD)	59 (16	59 (16)	64 (18)	55 (18)	68 (15)^*^
Anti-LGI1, mean (SD)		29 (20)	26 (11)	22 (11)	33 (21) ^*^
Anti-CASPR2, mean (SD)		63 (20)	57 (13)	53 (13)	68 (20)^*^
Anti-GABABR, mean (SD)		12 (22)	11 (12)	8 (11)	14 (25)
Anti-AMPAR1, mean (SD)		66 (20)	67 (14)	60 (16)	71 (19)^*^
Anti-AMPAR2, mean (SD)		6 (15)	4 (7)	3.5 (6.5)	6.8 (17)

* *p < 0.05*.

## Discussion

The results of the present study demonstrated that septic critically ill patients had autoantibodies against neuronal cell surface or synaptic proteins. Anti-NMDAR had an unexpected inverse association with hospital mortality but not with brain dysfunction.

The presence of autoantibodies against neuronal cell surface or synaptic proteins is probably secondary to the release of specific antigens by dying cells and its processing by memory B cells (1, 29). Izykenova et al. (30) reported that rats with induced cerebral ischemia exhibited increased plasma concentrations of NMDAR peptide fragments and antibodies to these receptor fragments (30). In humans, anti-NMDAR has been detected after ischemic stroke (8). Since these autoantibodies can have several pathophysiologic consequences, they may be not only biomarkers but may also participate in the development of a number of symptoms (4, 6, 13, 14, 17–21).

In the case of encephalitis, these autoantibodies, or B cells, cross the BBB to induce the prolonged synthesis of antibodies within the central nervous system (CNS) (1). The systemically-produced antibodies may reach the brain through a disrupted BBB (7). Outside of the content of critical illness, a synergism between anti-NMDAR and systemic lupus erythematosus-associated impaired cognition has been reported (31). Anti-NMDAR1 has been detected in individuals with slow cognitive impairment; the presence of this autoantibody affected synaptic protein expression and decreased NMDAR-mediated currents (32). Steiner et al. reported a higher prevalence of anti-NMDAR in schizophrenic patients (10). A seroprevalence of >10% was reported in a large-scale systematic screening for anti-NMDAR in the serum of healthy and neuropsychiatrically diseased subjects, many of whom were completely asymptomatic (33). In the context of critically ill patients, preoperative anti-NMDAR serum concentrations were predictive of severe neurological adverse events (delirium, transitory ischemic attack or stroke) after cardiac surgery with cardiopulmonary pass (13). It is uncertain if the persistence of these autoantibodies has any long-term clinical significance and should be followed-up, or if these patients are more prone to develop auto-immune encephalitis. None of the above cited studies presented a long-term follow-up to address this issue, and this should be evaluated further.

Brain dysfunction is a highly prevalent complication of sepsis (34) and is independently associated with increased short and long-term morbidity and mortality (35, 36). Additionally, BBB dysfunction is a hallmark of the development of sepsis-associated encephalopathy (37); thus, it is reasonable to suppose that autoantibodies are systemically produced during sepsis and could participate in the development of brain dysfunction. However, our results do not support this hypothesis. Additionally, the influence of autoantibodies on the function of organs other than the brain is not known. Since neurotransmitters are relevant not only to brain function in the context of sepsis (38–42), autoantibodies could also play a role in organ failure and mortality. Additionally, multiple organ failure and infections are one of the main causes of death in autoimmune encephalitis (43–45). Since some of these patients required ICU admission it is not possible to determine a causal link between the presence of autoantibodies and organ dysfunction. We did not observe a significant correlation between any of the measured autoantibodies and sepsis severity, organ dysfunction scores, and ICU mortality, suggesting that their presence did not have a direct pathophysiological role in sepsis progression. However, after adjusting for potential confounders, the fluorescence intensity of anti-NMDAR was inversely associated with hospital mortality. This is an intriguing factor that deserves further study. It is possible that their presence could be a surrogate to the general immune dysfunction, mainly T cell dysfunction, that occurs during sepsis development. Around 45% of septic patients were previously shown to have immunoreactivity to self-proteins, from KCNRG, a protein highly expressed in the lung and to autoantibodies associated with neurological targets (AQP-4 and GAD65) (22). The levels of some of these antibodies showed sustained elevation at days 20 to 28 after sepsis diagnosis. However, the clinical consequences of this observation are unclear. Additionally, due to our small sample size small changes in the number of events or non-events would have resulted in the loss of significance.

Several questions remain unanswered and the present study had several limitations. First, although none of the included patients had a clinical suspicion or laboratory abnormalities associated with autoimmune encephalitis, its presence cannot be completely excluded. Second, the titer of the antibodies was not defined, only the intensity of fluorescence, and a second technique such as enzyme-linked immunosorbent assay (ELISA) was not performed to confirm sample positivity. Third, in some conditions, such as after hospitalization for mania (12), there is a decrease in serum antibody positivity; we did not provide a time-dependent analysis of autoantibody levels after sepsis resolution. Fourth, the CSF levels of autoantibodies or BBB dysfunction were not assessed; this information could help to inform the relationship between autoantibodies and brain dysfunction in septic patients. Fifth, the relationship between autoantibodies and long-term cognitive impairment was not evaluated. This is a relevant topic for future study since, as seen in schizophrenic individuals, affected individuals with well-documented histories of birth complications and brain trauma show more severe neurological abnormalities when carrying anti-NMDAR (21). These findings strengthen the hypothesis of BBB involvement in these conditions and the long-term relationship between BBB dysfunction and cognitive impairment. Sixth, the number of measured events precluded the addition of more variables in the regression model to provide a more robust association between autoantibodies and outcomes.

In conclusion, anti-NMDAR was independently associated with hospital mortality but none of the measured antibodies were associated with brain dysfunction in septic patients. Studies including larger sample sizes are needed to confirm these results.

## Author Contributions

HM: protocol design, statistical analysis, manuscript preparation. IS: analysis of samples, statistical analysis, manuscript revision. JG: analysis of samples, manuscript revision. CaR and CT: collection of samples, manuscript revision. DG, FB, RW, FD-P, and CrR: protocol design, statistical analysis, and manuscript revision.

### Conflict of Interest Statement

The authors declare that the research was conducted in the absence of any commercial or financial relationships that could be construed as a potential conflict of interest.
