# Supplementary material for: Anti-NMDA Receptor Autoantibody Is an Independent Predictor of Hospital Mortality but Not Brain Dysfunction in Septic Patients
Source: Front Neurol. 2019 Mar 15;10:221. doi: 10.3389/fneur.2019.00221 (PMC6428735; doi:10.3389/fneur.2019.00221)

Figure 1 – Representative images of the fluorescence for (A) anti-NMDAR, (B) anti-LGI1, (C) anti-CASPR2, (D) Anti-GABABR, (E) anti-AMPAR1 and (F) anti-AMPAR2.

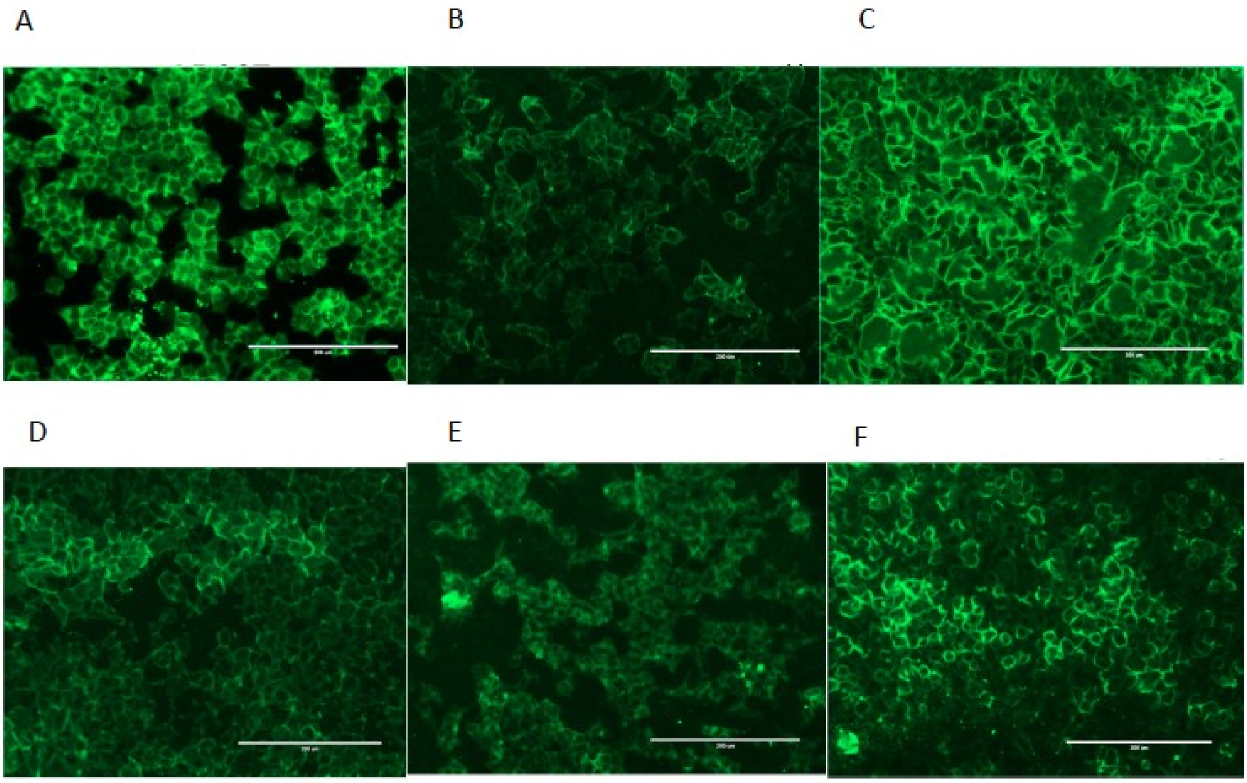

Supplement: Supplementary file 1 [file Data_Sheet_1.PDF]
